# Supplementary material for: Smartphone-Based Body Location-Independent Functional Mobility Analysis in Patients with Parkinson’s Disease: A Step towards Precise Medicine
Source: J Pers Med. 2022 May 19;12(5):826. doi: 10.3390/jpm12050826 (PMC9143184; doi:10.3390/jpm12050826)
Supplement: Supplementary file 1 [file jpm-12-00826-s001.zip › jpm-1716784/jpm-1716784-supplementary-sendto conversion.pdf]

Smartphone-Based Body Location-Independent Functional
Mobility Analysis in Patients with Parkinson's Disease: A Step towards Precise Medicine

**Diogo Vila-Viçosa [1], Mariana Leitão [2], Raquel Bouça-Machado [2,3], Filipa Pona-Ferreira [2], Sara Alberto [1],
Joaquim J. Ferreira [2,3,4] and Ricardo Matias [1,5,*]**

[1] Kinetikos, 3030-199 Coimbra, Portugal; dvicosa@kinetikoshealth.com (D.V.-V.); salberto@kinetikoshealth.com (S.A.)
[2] CNS—Campus Neurológico Sénior, 2560-280 , Portugal; marianaleitao.ft@gmail.com (M.L.); raquelbouca@cnscampus.com (R.B.-M.); filipaponaferreira@campus.ul.pt (F.P.-F.); joaquimjferreira@cnscampus.com (J.J.F.)
[3] Instituto de Medicina Molecular João Lobo Antunes, 1649-028 Lisbon, Portugal
[4] Laboratory of Clinical Pharmacology and Therapeutics, Faculdade de Medicina, Universidade de Lisboa, 1649-028 Lisbon, Portugal
[5] Physics Department & Institute of Biophysics and Torres VedrasBiomedical Engineering (IBEB), Faculty of Sciences, University of Lisbon, Campo Grande, 1749-016 Lisbon, Portugal
* Correspondence: rmatias@kinetikoshealth.com

Supplementary Materials

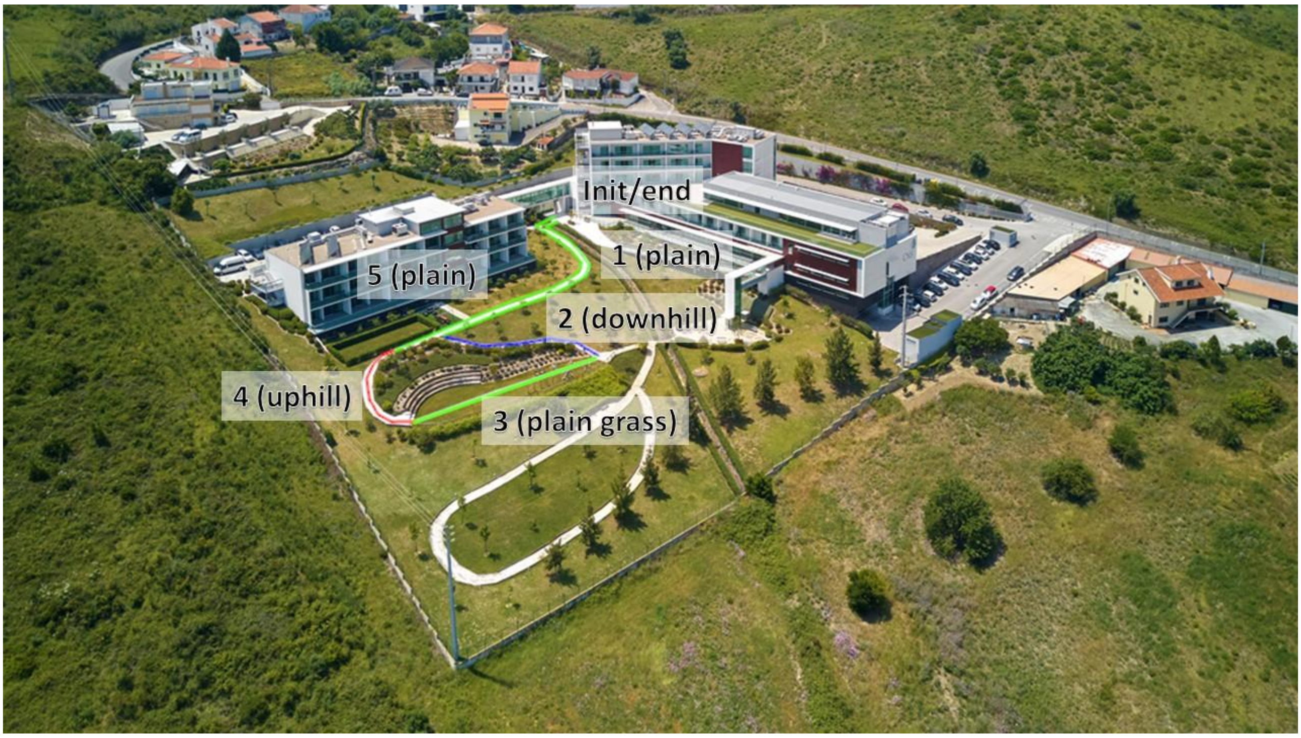

Figure S1: Global aerial view of the garden where the trial was performed.

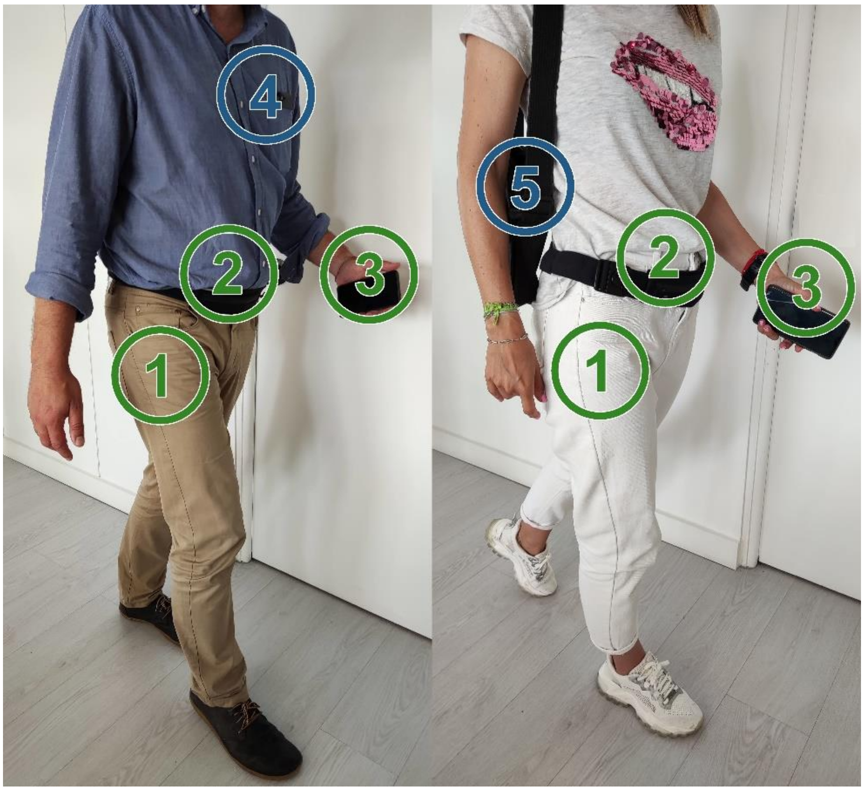

Figure S2: Representative smartphone locations: 1, 2 and 3 correspond, respectively, to Pants, Belt and Hand, which all 20 subjects carried. 4 and 5 correspond to Pocket and Bag, carried by 10 subjects each.

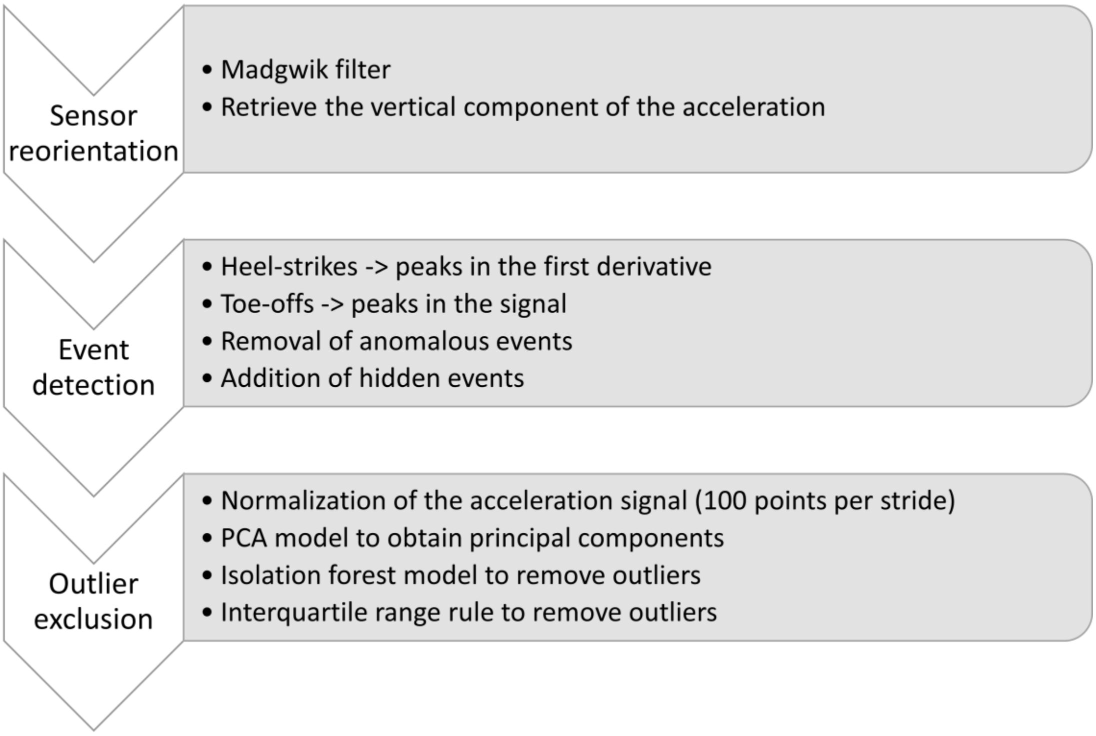

Figure S3: Pipeline of data processing comprising three main steps: sensor orientation, event detection and outlier exclusion.

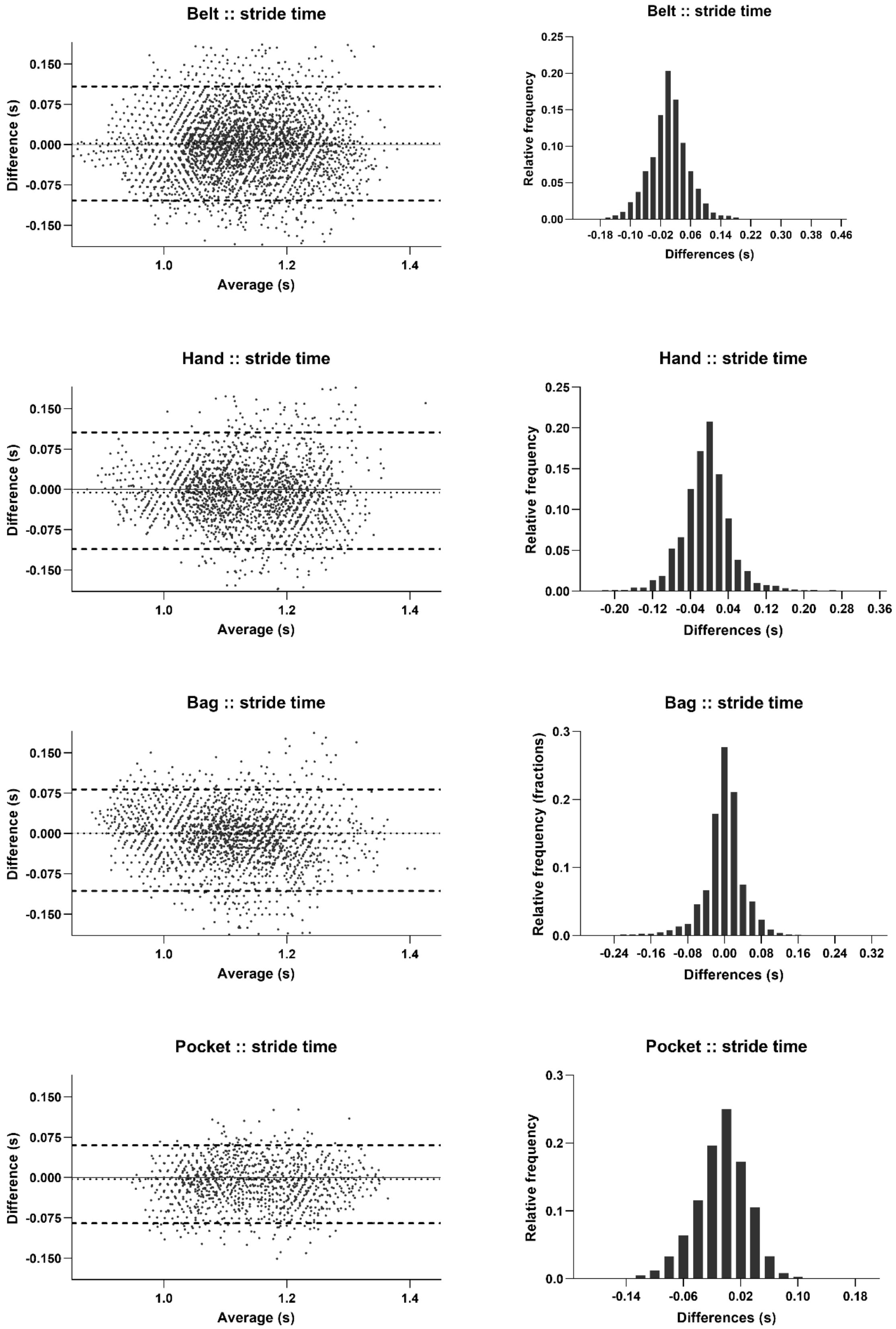

Figure S4: Bland-Altman comparison of the Pants stride time with those for all other locations. Dashed lines represent bias (median) and LoA (quantiles 2.5% and 97.5%).

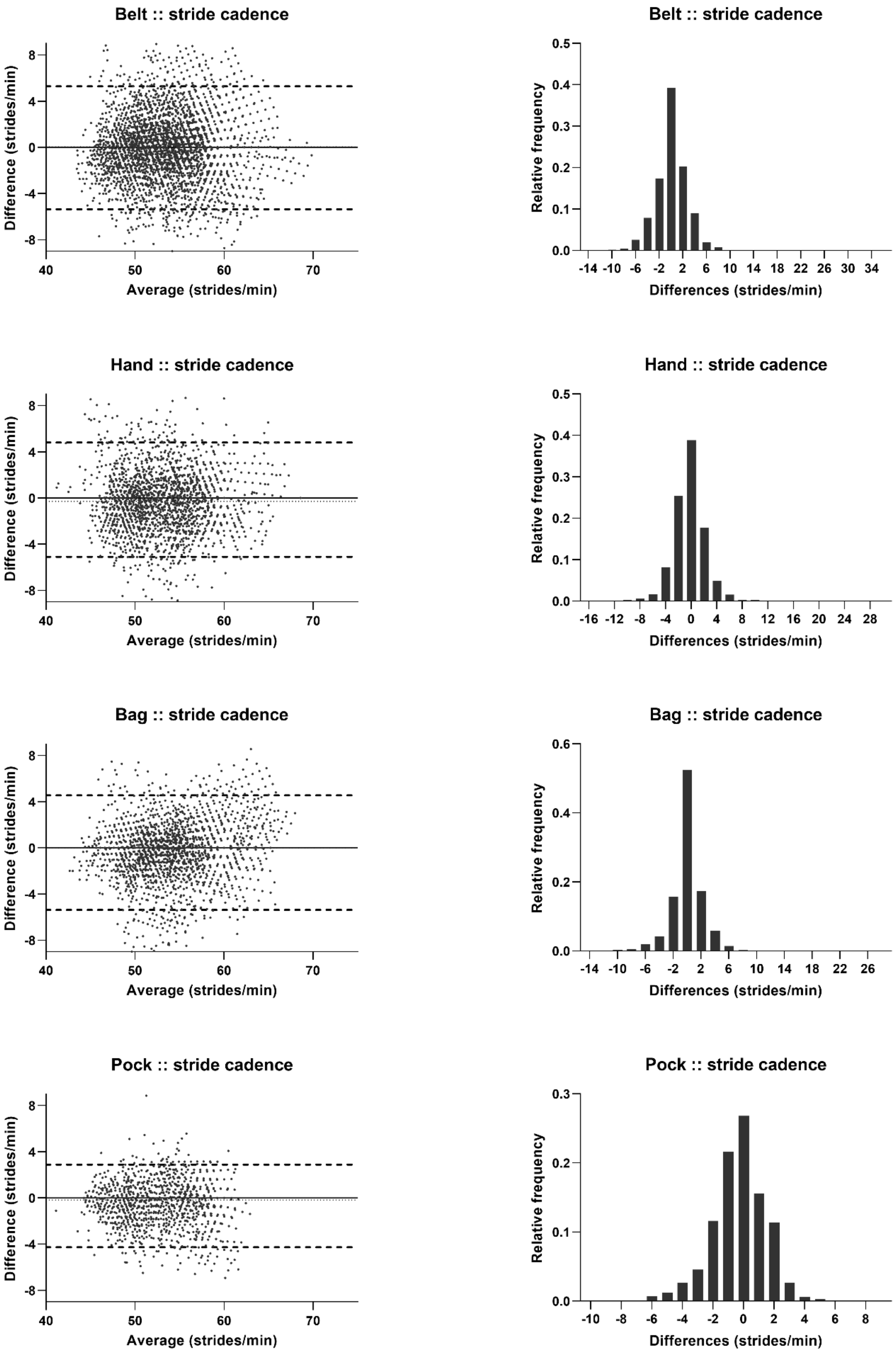

Figure S5: Bland-Altman comparison of the Pants stride cadence with those for all other locations. Dashed lines represent bias (median) and LoA (quantiles 2.5% and 97.5%).

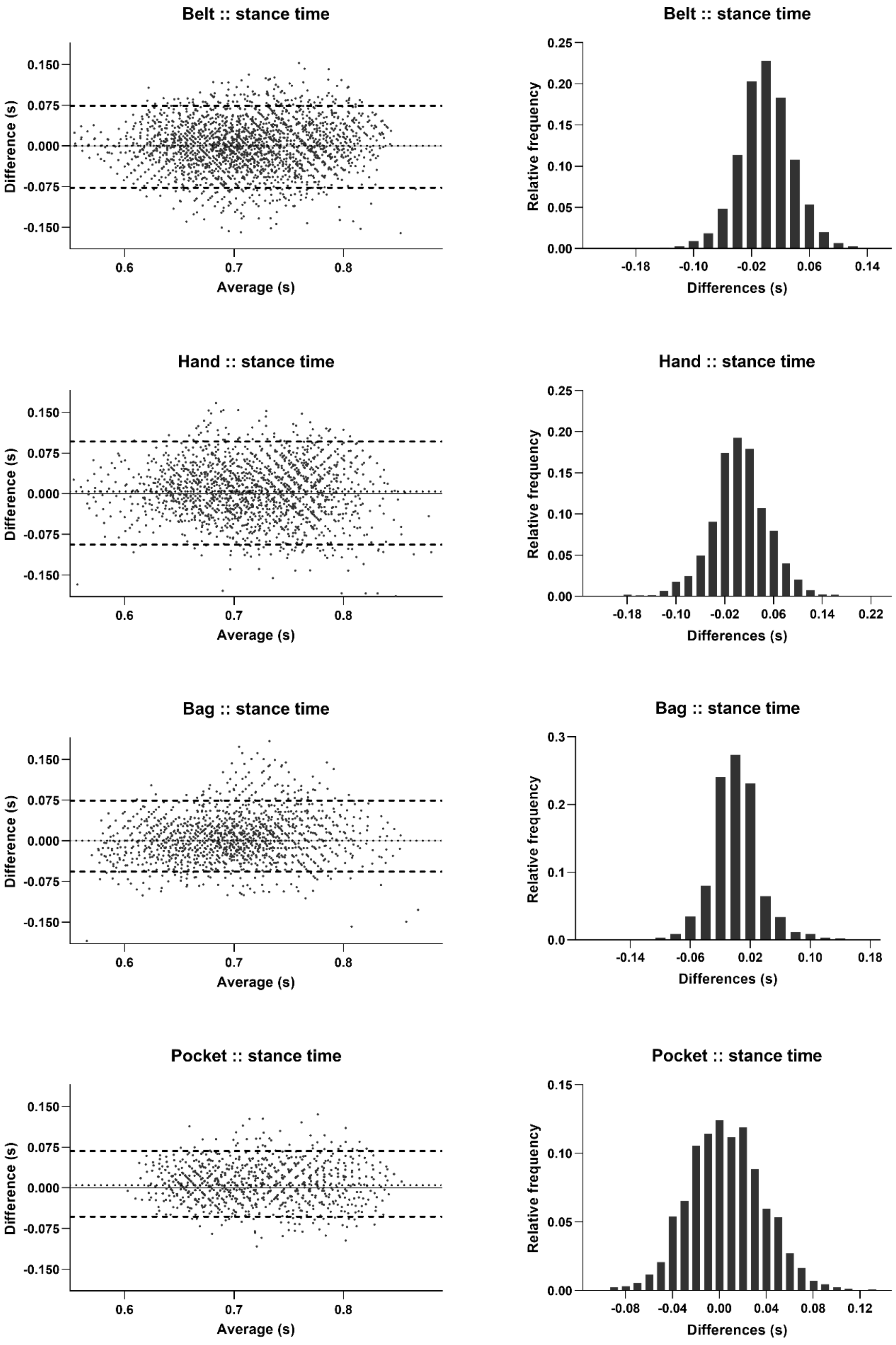

Figure S6: Bland-Altman comparison of the Pants stance time with those for other locations. Dashed lines represent bias (median) and LoA (quantiles 2.5% and 97.5%).

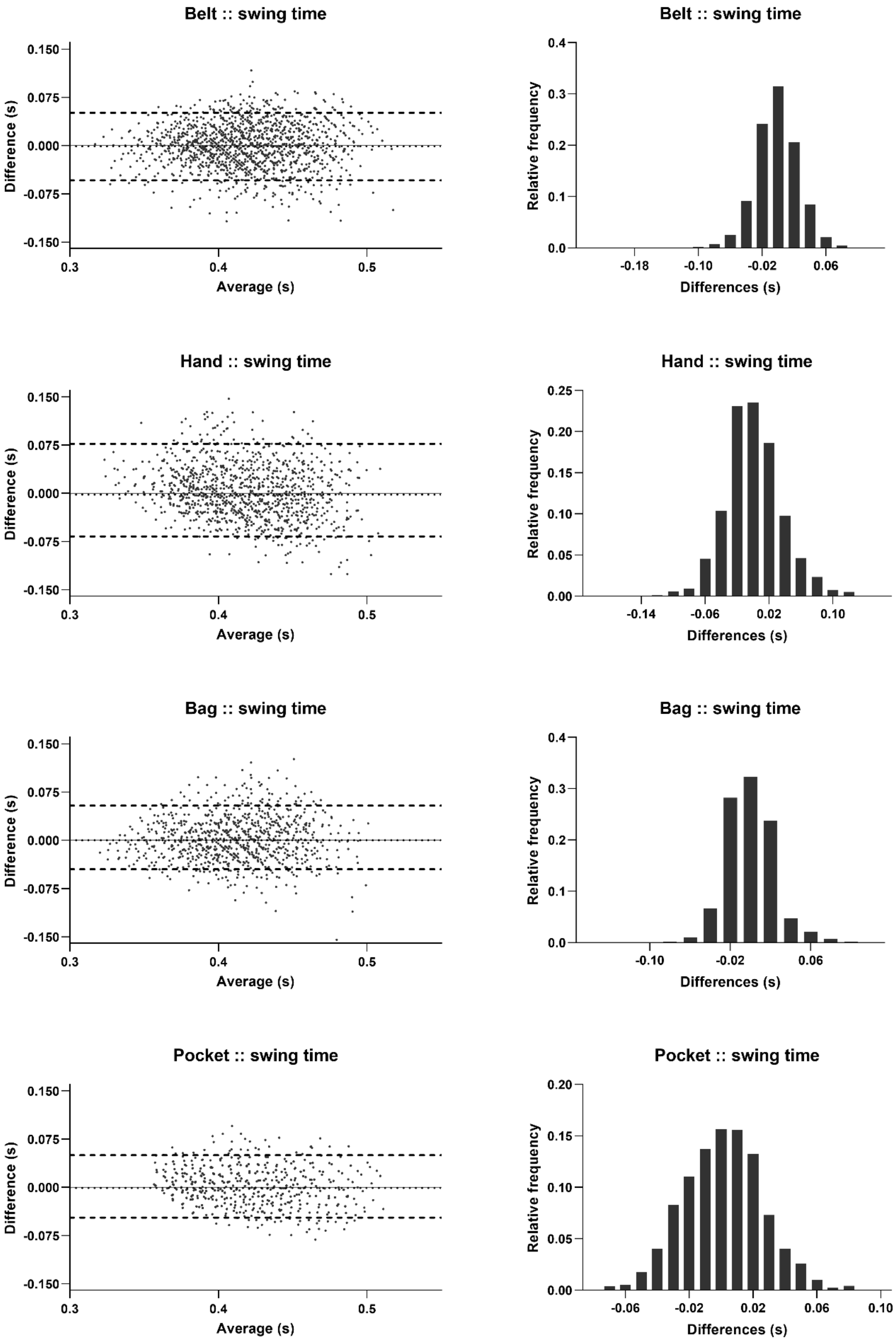

Figure S7: Bland-Altman comparison of the Pants swing time with those for all other locations. Dashed lines represent bias (median) and LoA (quantiles 2.5% and 97.5%).

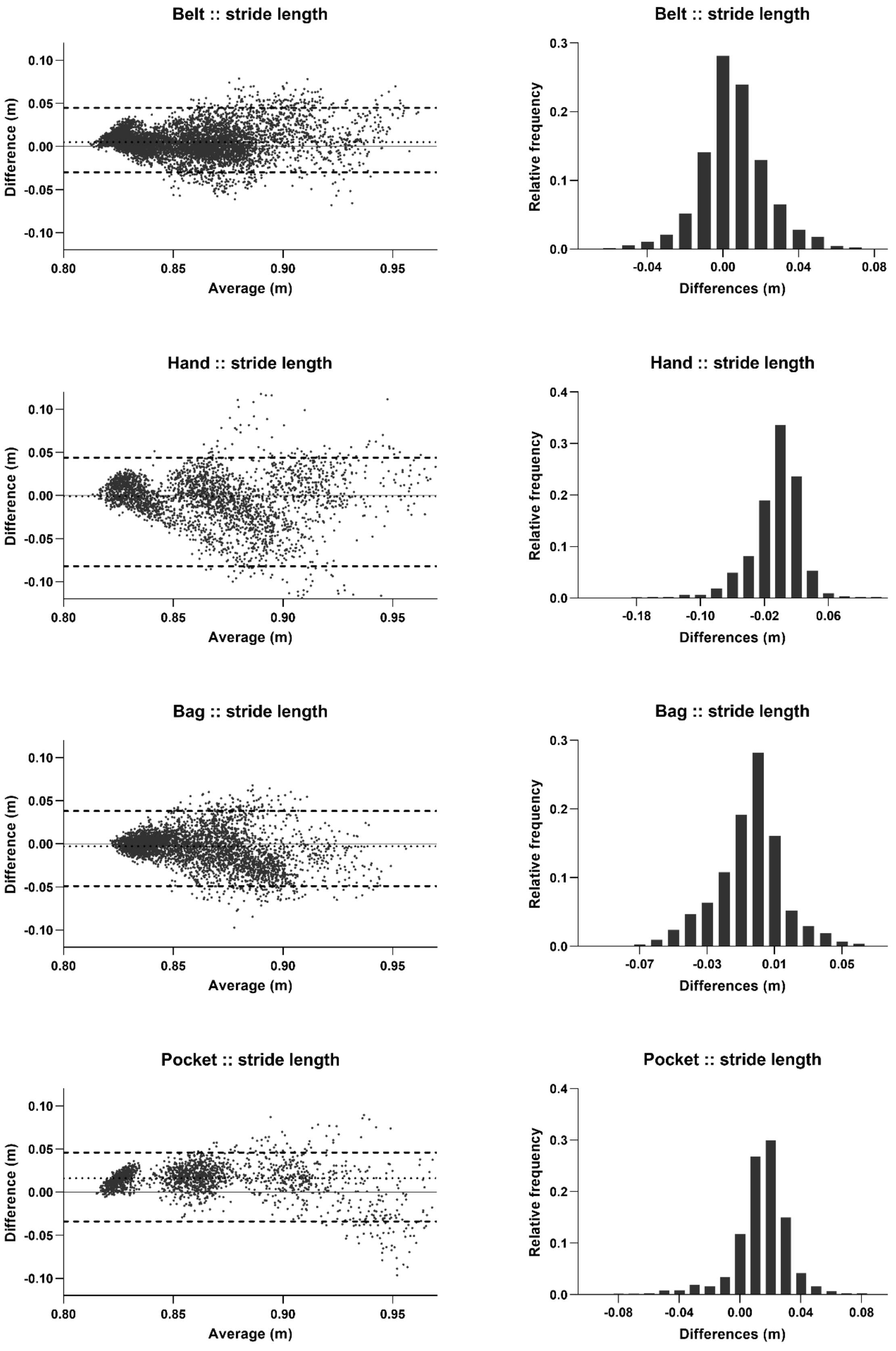

Figure S8: Bland-Altman comparison of the Pants stride length with those for all other locations. Dashed lines represent bias (median) and LoA (quantiles 2.5% and 97.5%).

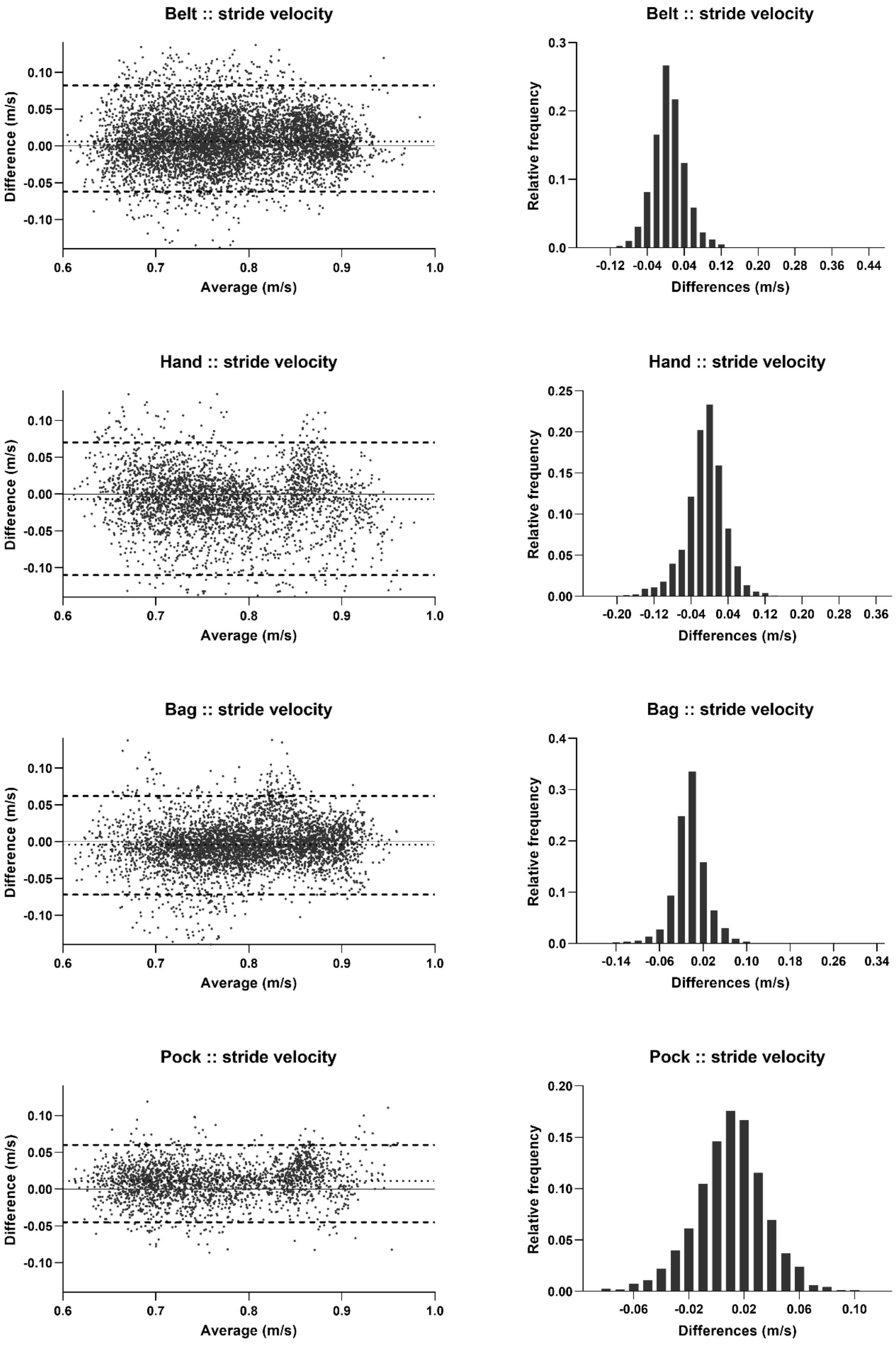

Figure S9: Bland-Altman comparison of the Pants stride velocity with those for all other locations. Dashed lines represent bias (median) and LoA (quantiles 2.5% and 97.5%).

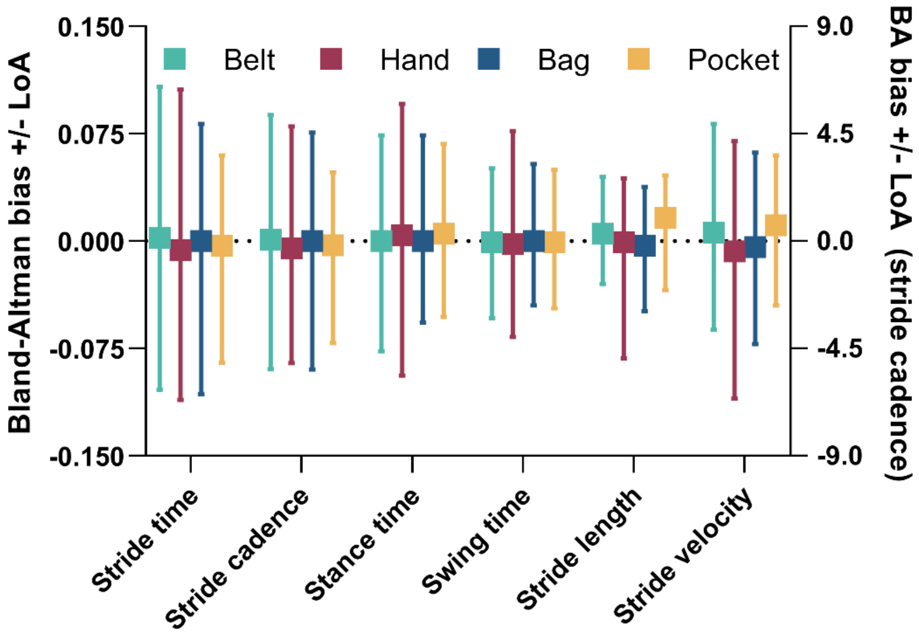

Figure S10: Agreement between gait parameters for Pants and for the other smartphone locations. Bias and LoA from the Bland-Altman analysis are shown.
